# Estimating exome genotyping accuracy by comparing to data from large scale sequencing projects

**DOI:** 10.1186/gm473

**Published:** 2013-07-31

**Authors:** Verena Heinrich, Tom Kamphans, Jens Stange, Dmitri Parkhomchuk, Jochen Hecht, Thorsten Dickhaus, Peter N Robinson, Peter M Krawitz

**Affiliations:** 1 Institute for Medical Genetics and Human Genetics, Charité Universitätsmedizin Berlin, Augustenburger Platz 1, 13353 Berlin, Germany; 2 Smart Algos, Retzbacher Weg 83, 13189 Berlin, Germany; 3 Department of Mathematics, Humboldt-University Berlin, Unter den Linden 6, 10099 Berlin, Germany; 4 Berlin-Brandenburg Center for Regenerative Therapies (BCRT), Charité Universitätsmedizin Berlin, Augustenburger Platz 1, 13353 Berlin, Germany; 5 Max Planck Institute for Molecular Genetics, Ihnestraße 63-73, 14195 Berlin, Germany

## Abstract

With exome sequencing becoming a tool for mutation detection in routine diagnostics there is an increasing need for platform-independent methods of quality control. We present a genotype-weighted metric that allows comparison of all the variant calls of an exome to a high-quality reference dataset of an ethnically matched population. The exome-wide genotyping accuracy is estimated from the distance to this reference set, and does not require any further knowledge about data generation or the bioinformatics involved. The distances of our metric are visualized by non-metric multidimensional scaling and serve as an intuitive, standardizable score for the quality assessment of exome data.

## Background

In recent years, next-generation sequencing (NGS)-based exome screens have become an invaluable tool in Mendelian disease gene discovery and are now being introduced as clinical diagnostic tools for genetic disorders of high phenotypic and genetic heterogeneity [1,2]. Various solutions for exome enrichment and sequencing exist and numerous algorithms for sequence read mapping and variant detection are in use [3-12]. There are recommendations for sequencing depth and benchmarks for the distribution of sequence read coverage over the target region. The common core of the diverse approaches to sequence exomes represents the consensus coding sequences as defined by the consensus coding sequence (CCDS) project [13]. The majority of publications [12] related to this field seem to confirm that a mean sequencing depth of this target region with high quality short sequence reads should be above 50-fold and more than 90% of the CCDS exons should be covered by at least 10 sequence reads for diagnostic purposes. Another recently introduced parameter for the quality assessment of multiple read alignments is the variance of the ratio of reads that support the alternate allele at heterozygous positions [14]. The lower this variance, the lower the error rate to be expected from amplification artifacts. The ratio of transitions versus transversions (ti/tv) and the proportion of variants that are already listed in databases of genetic variation such as the Single Nucleotide Polymorphism database (dbSNP) [15] are measures of quality that may be applied to the entire variant call set of an exome. The ti/tv ratio should be close to 3:1 for the CCDS exons, and the proportion of singletons should be below 10% [16]. However, the ti/tv ratio is influenced by the target region, whereas the number of novel variants may depend on the background population. The higher the amount of non-coding variants, the lower the ti/tv ratio, and higher proportions of novel variants may be observed if the sequenced individual is from a population that is poorly represented in the variant databases.

Although these parameters may serve as valuable indicators for quality they do not directly indicate the accuracy of a sequenced exome and to our knowledge there is no criteria for assessing whether the variants identified by whole exome sequencing represent a comprehensive list. Specifically, it is not possible to estimate an exome-wide false positive or negative rate for variant detection that is purely based on the quality scores of genotype calls. Sequencing technology-specific error signatures [17] can yield artificial variant calls of erroneous high quality and result in an underestimated proportion of false calls, while poorly adjusted bioinformatics pipelines for data processing may lead to missed calls. In most NGS studies, a Phred-like quality score is provided for each called genotype. This score describes the confidence in a genotype call. Based on a certain likelihood model for genotypes, the Phred score represents the probability that the genotype call is wrong, given the reads in an alignment $\left(\text{Phred score = }-10log_{10}Pr\left(wrong\;genotype\right)\right)$). In a model that assumes, for example, a Bernoulli distribution of the sequence reads at a heterozygous position, the Phred score of a heterozygous genotype would decrease the more the ratio of reads supporting the alternate allele deviates from the expected value of 0.5. However, this quality score not only depends on the raw data but also on the mapping algorithms and probability models that were used for variant calling. That means that processing the same raw data by different bioinformatics pipelines may result in different distributions of quality scores, suggesting different genotyping error profiles for the same exome. Even variant calling approaches that are based on similar Bayesian methods do not yield the same genotype probabilities due to different priors [18], and methods of quality score recalibration cannot completely adjust for that effect (Additional file 1, Table S1).

In order to enable interoperability and platform independence, we have developed a method to measure the accuracy of a set of variants by assessing its composition. In our metric, the distance between sets of variants from two exome samples is computed without considering genotyping quality scores. The basic idea is that the distance between variant sets of comparable quality is closer than the distance between variant sets of very different quality. In our comparison, the variant data of individuals of the 1000 genomes project [19] serve as a gold standard and we refer to them as the reference set. If the genotype concordance between the reference variant call set and the test variant call set is high, this suggests a comparable sequencing quality. We will show in the following that the distance of a test sample to this high quality reference data is an indicator for the genotyping accuracy of the exome.

## Methods

### Generation of exome data, accession of reference data and data processing

Genomic DNA of European individuals was enriched for the target region of all human CCDS exons with SureSelect Human All Exon Kit (Agilent, Santa Clara, USA) according to the manufacturer's protocol and sequenced on a HiSeq 2000 (Illumina, San Diego, USA), yielding more than 5 gigabases raw sequence data per exome. The Charité University Medicine ethics board approved this study, which conforms to the Helsinki Declaration, and we obtained informed consent of all participants.

Publicly available NGS raw data and variant calls of 1,063 individuals of different populations were downloaded from the ftp server of the 1000 genomes project [19]. Exome variants of these individuals served as reference variant sets in our work. Exome variants of the 5000 exomes project and of de Ligt *et al*. were used for testing the accuracy predictions [20,21].

Exomes of test samples were enriched with Human All Exon SureSelect baits from Agilent and sequenced on Illumina Genome Analyzer IIx and Illumina HiSeq 2000 as 100 bp single-end reads or paired-end reads according to the manufacturers' protocols. Short sequence reads were mapped by Novoalign (Novocraft, version 2.08) or BWA[22] to the reference sequence GRCh37. Variants were detected with default settings with SAMtools [23] or GATK [10] on bam-formatted alignments [22]. Variant calls in variant call format (vcf) [24] were restricted to single nucleotide changes and to the consensus exome target region of the 1000 genomes project. Additionally, sites that were classified as technical artifacts by the 1000 genomes project were ignored.

### Distance function

The distance $d_{ij}$ between any two samples $x_{i}$ and $x_{j}$ for all positions  k in the target region (exome), where the called genotypes differ from the reference sequence in at least one sample, can be calculated by a weighted indicator function $I\left(x_{i}\left(k\right),x_{j}\left(k\right)\right)*W_{ij}\left(k\right)$, with:

$I_{ij}\left(k\right)=I\left(x_{i}\left(k\right),x_{j}\left(k\right)\right)=\left\{\begin{array}{c} 1,if\;x_{i}\left(k\right)==x_{j}\left(k\right)\\ 0,if\;x_{i}\left(k\right)\neq x_{j}\left(k\right) \end{array}\right)$

and $W_{ij}\left(k\right)=\frac{2}{f\left(x_{i}\left(k\right)\right)+f\left(x_{j}\left(k\right)\right)}$.

This means that for the same genotypes the indicator *I *is weighted by the reciprocal of the genotype frequency $f\left(x_{i}\left(k\right)\right)$, which is based on the reference set with an appropriate background population. To give an example, a genotype for individual  i at a given position  k, $x_{i}\left(k=chr6:79595096\right)=C/C$, would refer to a genotype frequency $f\left(x_{i}\left(k\right)\right)=0.999$, if 1 out of 1,000 individuals in the reference set differs from this genotype.

For genotypes that were present only in the test sample but not observed at all in the reference set, we set their frequency to1/(n+1), where  n is the total number of individuals in the reference set.

Based on that the distance $d_{ij}$ is defined as:

$$d_{ij}=1-\frac{1}{C_{ij}}\underset{k}{\sum }I_{ij}\left(k\right)*W_{ij}\left(k\right)$$

where $C_{ij}=\underset{k}{\sum }W_{ij}\left(k\right)$ is used as a normalizing constant.

Therefore, a disagreement at a position of low variability in the reference set contributes more to the total distance than one at a highly variable position.

In the resulting distance matrix,  D, pairs of individuals who are 'closely related' can be distinguished from those who are distinctly apart by lower distance values. Thus, a distance $d_{ij}=0$ means total agreement of all genotypes and a distance value of $d_{ij}=1$ means total disagreement of all genotypes.

### Visualization of distance matrices by non-metric multidimensional scaling

The output of the above-described pairwise comparison of variant sets is a high-dimensional distance matrix with given distances or dissimilarities between pairs of individuals that satisfy all conditions of a metric. To represent the dissimilarities as distances between points in a low-dimensional space, we used a statistical technique named non-metric multidimensional scaling (MDS), that is, a visualization method such as principal component analysis or metric MDS. However, in contrast to principal component analysis (PCA) and metric MDS, non-metric MDS does not make any assumptions about the distribution of the underlying high-dimensional data. With a pre-specified number of dimensions for the embedding φ and an appropriate initial configuration, the *isoMDS *function of the *MASS *R-package was used to minimize the goodness of fit, called stress *S*, of Kruskal and Shepard (see [25]). To promote readability and an easy interpretation of the data, we chose a standard two-dimensional embedding with:

$$S(x_{1},\ldots ,x_{n},\varphi )=\sqrt{\frac{\sum_{i=1,i\neq j}^{n}{\left(d_{ij}-||\varphi (x_{i})-\varphi (x_{j})||\right)}^{2}}{\sum_{i=1,i\neq j}^{n}\left||\varphi (x_{i}\right)-\varphi (x_{j})|{|}^{2}}}$$

where ||⋅|| defines the Euclidean norm.

### Down sampling of raw data and simulation of genotyping accuracy

For coverage-adjusted comparisons, we randomly removed sequence reads from the original alignments. Variants were recalled on these down-sampled exomes as described above. As genotyping accuracy we define the percentage of the entire exome that was correctly genotyped, that is the sum of true positive genotype calls (alternate and reference genotypes) divided by the entire size of the exomic target region. For our simulations, we assumed that the reference set had a genotyping accuracy of 100% and introduced genotyping errors at random positions. As most of the exomic positions had low variability in the reference set, the contribution of genotyping errors to the distance function could be approximated by adding twice a binomial distributed random variable, $X\sim B\left(N,p\right)*2$, to the normalizing constant $C_{ij}$, with probability *p *equaling the specified genotyping error and the number of trials $N=2.8*10^{7}$bp is the total size of the exome,.

### Computation of the standardized dissimilarity score and reference curve

Distances between all individuals of the reference set were measured and the averaged values of the median and interquartile range of all columns of the distance matrix were computed to standardize the median of a test sample. The median of the distances from a test sample to all individuals of the reference set was computed and normalized by subtracting the pre-calculated median of the reference set and dividing the interquartile range (IQR) of the reference set. The reference curve and both 5% and 95% quartiles for the standardized dissimilarity score (SDS) were computed for the reference set and simulated data sets of decreasing error groups.

## Results

### An error sensitive genotype-weighted metric

Like any metric, the distance measure that we used to compare different sequences of a set of test samples induces a topology. Variant calls, which describe the measurable differences between samples, represent true genetic variation, as well as genotyping errors. The subject of our work is the quality assessment of a set of exome genotypes, thus our metric needs to induce a topology that is sensitive to sequencing errors while being robust to true genetic differences. By using a weighting method for genotypes that uses their frequency of occurrence, we achieved higher precision in accuracy prediction compared to an unweighted hamming distance (Figure S1 in Additional file 1). If two samples are not the same at an exonic position, which is highly constrained in the population, this contributes more to the total distance, because such an event has a higher probability of being a genotyping error than divergent genotypes at a highly variable site of the exome. When we compare two exomes, our metric works on all genotypes that have been called in these samples. The genotypes are weighted by the degree to which the genotype is constrained in the population. Though several definitions for measuring the degree of genotype conservation have been suggested [26,27], most variable positions in the human genome are biallelic and for simplicity we approximate the conservation of a genotype by the inverse of its frequency. Thus the differences in two variant sets are weighted by their respective genotype frequencies, and the detection of many rare variants in a test sample therefore points to a higher proportion of false positive genotype calls. By contrast, if many variants that are common in the population are not detected in a test sample, this points to a high false negative error rate. By this means, we compute a matrix that contains the distances of the test sample to all the samples of a reference set as well as their mutual distances. These distances are a result of a function that works on the entire exomic target region, as defined by the 1000 genomes project, and may therefore be viewed without distortion only in the multivariate, exomic space. We have implemented and tested our method using whole exome data, but it could be applied to other types of high-throughput sequencing data. However, the precision of predicting the accuracy of genotyping decreases for smaller target regions (Figure S2 in Additional file 1).

### Non-metric multidimensional scaling is best suited for distance visualization

Because distance is based on multiple variables of weighted categorical data, visualization in a plane requires a transformation. We tested several standard techniques of data visualization and found that non-metric MDS [28] showed the best characteristics in conveying the differences in genotyping accuracy in two dimensions (Figure S3 in Additional file 1). We therefore project the exomic distance matrix into two artificial dimensions of Φ1 and Φ2 that have the smallest loss of information [29-31].

**Figure 1:**
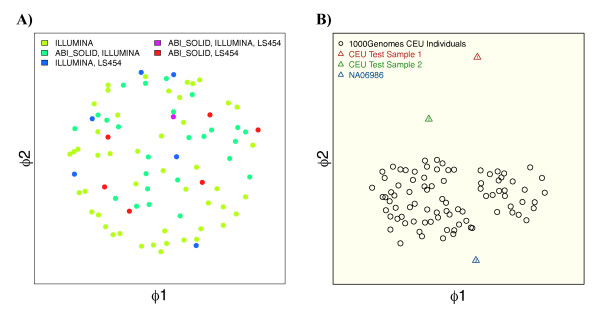
**Similarities of exome samples**. **(A) **The similarity between exome samples is measured with a genotype frequency weighted metric. The similarity matrix is visualized by non-metric multidimensional scaling in the two-dimensional plane. Variant sets of individuals of European descent that were analyzed by the 1000 genomes project form a homogeneous cluster, irrespective of the applied sequencing technology. **(B) **Two test samples of the same ethnicity that we genotyped according to a standard exome protocol are compared to samples of the reference set. The larger distance of sample 1 to the reference cluster indicates a lower genotyping accuracy, while sample 2 points to a high quality.

The reference samples of the 1000 genomes project form clusters according to their ethnicity (Figure S4 in Additional file 1) and for any test sample we chose the closest cluster as the best matching reference set. Samples from the same population background form homogeneous clusters in non-metric MDS scaling, indicating a comparable genotyping quality (Figure 1A).

We then analyzed two test samples of European descent but of unknown genotyping accuracy and included them into the MDS projection of all central European (CEU) individuals from the 1000 genomes project, which is shown in Figure 1B. Except for one representative recalled sample, NA06986, all individuals of the CEU reference set are displayed as black circles, whereas the two exome test samples are represented by the colored triangles. Although the mean sequence coverage of the exome target region is above 60× for both test samples, they clearly differ in their mean distance to samples from the reference set: the second sample is close to the cluster formed by the individuals of the reference set, whereas the first sample is an outlier, indicating inferior quality. This considerable difference in the mean distance is remarkable given the high sequencing depth and a comparable ti/tv ratio of 3:2 (Additional file 1, Table S1). Also the proportion of variants found in dbSNP is around 97%, which is comparable to NA06986. Only the variance of the heterozygous allele frequencies, which increases with a growing number of artifacts from the amplification steps during the library preparation, suggests a lower quality for sample 1 with var(het AF) = 0.017 compared to 0.012 in test sample 2 and 0.013 in NA06986 at the same mean coverage [14].

### Visualization of exomes of different genotyping accuracy

**Figure 2:**
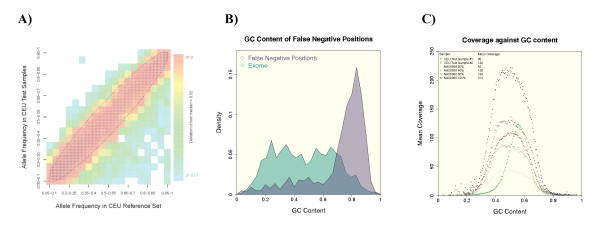
**Analysis of low quality samples**. **(A) **Comparison of variant allele frequencies in different sample sets. Value pairs of genotype frequencies were computed for exomes of the reference set (CEUs from the 1000 genomes project) and compared to test samples of the same ethnicity that are low quality. The ellipse indicates twice the standard deviation assuming a binomial model for the allele frequency *p*. Variants in the right lower quadrant were called with a lower probability in our test samples and are characterized by a GC content that deviates from the expected mean. **(B) **GC content at false negative positions. Variants that are underrepresented in exomes with a large distance to the high quality reference set are overrepresented in exome regions with high GC content (violet curve). The green curve indicates the distribution of the GC-content that is expected for an equal number of variants that are randomly drawn from the exome. **(C) **Coverage against GC content. The mean sequence coverage of the consensus exome varies with the GC content of the target region. The overall coverage for an exemplary sample from the 1000 genomes project (NA06986) was higher compared to test samples 1 and 2. Test sample 1 has a particularly low coverage in regions with extreme GC content, suggesting a higher error rate.

We measured the mean distance to the reference set for 85 exomes of European descent that were all analyzed in the context of NGS research projects. The two samples displayed in Figure 1B illustrate the extreme spectrum of the mean distance to the reference set that we encountered. We hypothesized that variants often detected in exomes of the 1000 genomes project, but not in the outliers of our test samples, might point to a subset that requires high data quality to be properly detected and that might explain partly the separation from the reference cluster. Figure 2A displays the value pairs of variant allele frequencies that are based on 85 CEU individuals from the 1000 genomes project and 85 exome test samples. For technical replicates one would expect all allele frequency value pairs to lie close to the diagonal in this kind of visualization. For two sample sets of equal size that are drawn from the same population, one would expect a certain degree of variance in the measured allele frequency that is simply based on the finite sample size: given the allele frequencies one would expect for a sample size of 85 that about 95% of the frequency value pairs fall inside the boundaries of the displayed ellipse based on a Bernoulli distribution. However, in our case there are considerably more outliers than expected by chance.

### Characteristics of sequence variants with high error rates

We looked for similarities of these outliers and computed the GC content of 100 bases flanking the variant alleles that were present in more than half of the individuals analyzed in the 1000 genomes project but in only one or less of our analyzed samples. The distribution of the GC content of these variants clearly deviates from the distribution that one would expect for randomly located variants in the exome (Figure 2B).

To investigate the reasons for the higher false negative error rate for variants in a GC-rich sequence context, we computed the mean read coverage of the target region depending on the GC-content. Figure 2C shows that the distribution of the coverage is smaller for test sample 1 compared to test sample 2 and NA06986 that was sampled down to a comparable mean coverage. Thus, the higher distance of test sample 1 to the reference set is partly due to a critically low sequence read coverage of regions with an extreme GC-content. Benjamini *et al*. studied the bias caused by GC content depending on read coverage in detail for Illumina sequencing data and showed that it also varies between technical replicates [32]. This also means that two samples may have different genotyping accuracy for the whole exome although they have been processed by the same protocol.

### The distance to the reference set versus coverage and error rates

**Figure 3:**
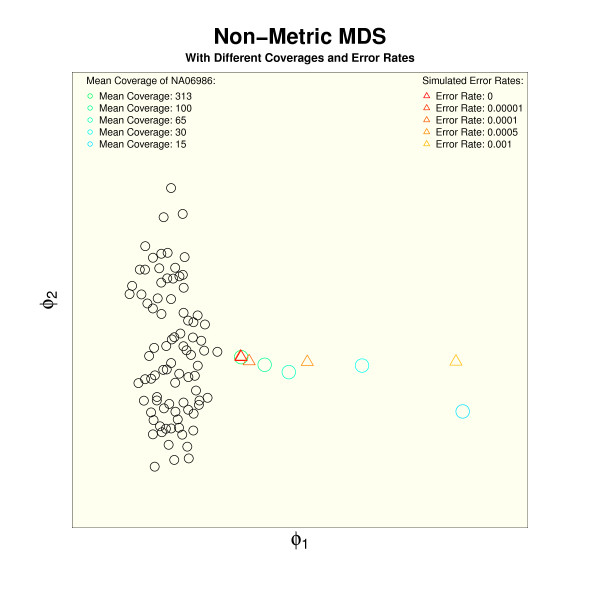
**Distance to the reference set for decreasing coverage and increasing error rate**. A reduction of the raw sequence amount for a randomly chosen sample of 1KG project, NA06986, reduces the similarity to the reference set (green-blue circles) indicating decreasing genotyping accuracy. A similar effect is observed when simulated genotyping errors are added to the variant calls (red-yellow triangles). A decreasing similarity of the test samples results in divergent trajectories.

We hypothesized that the distance to the high quality reference set should grow when the amount of raw sequence data decreases. We therefore successively reduced the sequence coverage in the raw exome data of NA06986, called variants anew, and observed an increasing distance to the reference set (green to blue circle in Figure 3). A decreasing sequencing coverage will not only yield an increasing rate of false negative genotypes but also increase the rate of false positive calls. It is more likely that a sequencing error will be called as a heterozygous variant, and a heterozygous variant as homozygous, if the position is only covered by a few reads. We then analyzed how an increasing false positive error affects the distance to the reference set by simulating detection artifacts that were randomly distributed over the target region and added to a sample from the reference set. The triangles in Figure 3 show that the data points follow a trajectory that departs from the reference cluster with growing error rate. It has to be noted that the visualization of multiple versions of the same test sample that differ only in coverage or error rates in a single MDS plot contorts the relative distances. All the depicted simulated data points in Figure 3 originate from the same data set and are therefore self-similar. The self-similarity is high for a low error rate and high coverage and decreases with increasing error rate and decreasing coverage, as the divergent trajectories of circles and triangles indicate. We also obtained similar results in the analysis of simulated data sets of other ethnic backgrounds (Figure S5 in Additional file 1).

A genotyping error of 0.00001 corresponds to an expectation of one genotyping error in approximately 100 kb of the target region. Two randomly chosen samples from the reference set would differ in about 100 positions in such a window of 100 kb [21]. The samples with the simulated error rate begin to separate from the high quality cluster for error rates above 0.00001, which corresponds to a positive predictive value of 0.99 (number of true positive divided by number of positive calls). Interestingly, the positive predictive value that was reported by Tennessen *et al*. for the variant calls of the 5000 exomes project is between 0.97 and 0.98 [21]. The resolution of our visualization techniques is therefore sufficient to display these qualitative differences.

### Comparison of exome data from different next-generation sequencing studies

**Figure 4:**
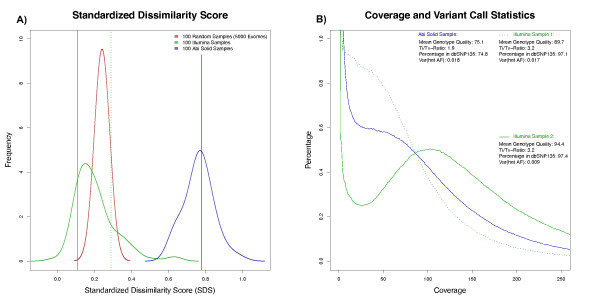
**Distributions of standardized dissimilarity scores for different exomes**. **(A) **100 samples, including test samples 1 and 2, were sequenced on the Illumina platform with mean coverage above 60×. The mean SDS for the Illumina samples is comparable to the mean SDS of 100 exomes that were simulated based on genotype frequencies from the 5000 exomes project. The variation of these SDS is smaller due to missing haplotype information. (B) The mean SDS of 100 exomes that were sequenced on a Solid platform is considerably higher due to a lower and less uniform sequence distribution over the target region and due to less accurate variant calls.

In contrast to the 1000 genomes project, the genotype calls from the 5000 exomes project were publicly available only in a collapsed form as genotype frequencies for European Americans and African Americans and not as separate variant sets for each sample. In addition to our in-house exome data, we also analyzed the distances to the reference cluster for exomes that we simulated based on the genotype frequencies from these European Americans and 100 exomes that were already studied by de Ligt *et al. *[20]. Figure 4A shows the distribution of the SDS, which represents a normalized distance of a test sample to the reference cluster. The mean SDS for the simulated exomes of the 5000 exomes project is comparable to our exome data and lower than the SDS of the de Ligt *et al*. exomes. The smaller variance of the SDS distribution in the 5000 exomes samples, which points to a higher self-similarity, is due to a simulation process that did not properly represent the haplotype substructure of the data. The higher mean SDS of the de Ligt *et al*. data is mainly explained by outdated genotyping algorithms with higher error rates and a lower mean coverage of the target region. Figure 4B depicts the coverage distribution over the exome and additional quality parameters for an exemplary sample from de Ligt *et al*. and our two test samples.

**Figure 5:**
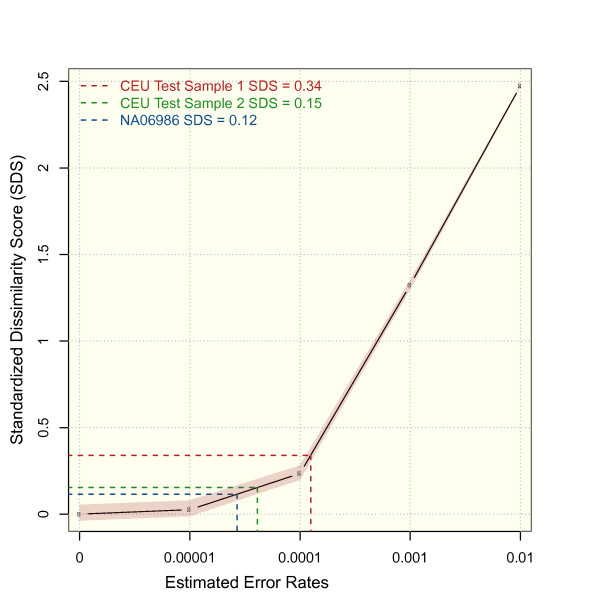
**Estimation of genotyping errors from standardized dissimilarity scores**. The reference curve with its 5% and 95% quantiles is based on the distances of samples with simulated error rates to the reference set. The SDS of a test sample indicates its error rate by its intersection with the reference curve. The estimated error rate of test sample 1 is considerably higher than of test sample 2 and of NA06986 from the 1000 genomes project. SDS, standardized dissimilarity score.

By simulating increasing sequencing noise for all exome data sets of the 1000 genomes, we derived distributions for the mean distances of the original data. Based on these distributions, we computed a reference curve for the SDS of an unknown sample that correlates with its exome-wide accuracy (Figure 5). The SDS that is measured for our test samples may be used for estimating their genotyping accuracy by intersecting with the reference curve. For test sample 1, the mean distance to the reference set was 0.29, which corresponds to an estimated genotyping error of 0.0001. By contrast, the SDS of 0.11 for test sample 2 indicates an error rate that is much closer to that of the 1000 genomes project. Interestingly, the distance to the reference set shows characteristics of a phase transition, when the contribution of genotyping errors exceeds the genetic variability between individuals. We also checked the validity of our approach by deriving the genotyping accuracy via a complementary method. In Heinrich *et al*., we analyzed the effect of amplification steps during sample preparation and derived rates of genotyping errors from technical replication [14]. The SDSs for these replicates indicate genotyping accuracy for the exomes between 99.99% and 99.999%, which is in good agreement with the previously computed accuracy.

Thus, the SDS is a parameter derived from the composition of a variant set and is even more powerful in predicting the data quality than other quality control parameters such as coverage distributions, which require access to the read alignments (Additional file 1, Table S1).

**Figure 6:**
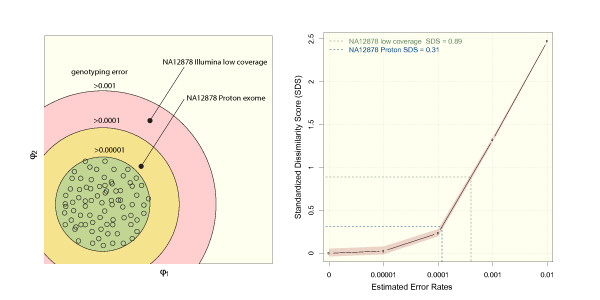
**Comparison of exome data of different NGS platforms**. A) Exome genotypes of individual NA12878 were called based on an Illumina read data set of low coverage and a Proton dataset. The Proton exome is closer to the CEU reference data than the low coverage Illumina exome. B) The accuracies of the exome genotype sets were estimated based on the mean distance to the reference set.

We tested the influence of the sequencing platform on the error prediction by our approach by restricting the reference set to samples that were sequenced with the same technology (Illumina). Although the visualization in metric MDS clearly shows that the test samples are closest to the Illumina samples from the reference set (Figure S3 in Additional file 1), the effect of the sequencing platform on the accuracy prediction is marginal (Figure S6 in Additional file 1). The SDS is therefore robust and can be applied to predict the quality of genotyping data from different sequencing technologies. We used the platform independence of our approach to analyze the quality of a sample from the 1000 genomes project, NA12878, that was recently re-sequenced by a new technology (Proton, Ion Torrent, aligned with TMAP and genotyped with variantCaller). In Figure 6, the distance of this Proton variant set of NA12878 to the reference set is compared to a variant set of the same individual based on Illumina raw data that was down-sampled to a mean coverage of 30 fold as described above. This figure visualizes how the quality of two exomes of the same individual that were generated on two different sequencing platforms and processed by different bioinformatics pipelines may be interpreted at a glance. We used the SDS to estimate the genotyping accuracy and predicted 99.9% for the low coverage Illumina exome and above 99.99% for the Proton exome, which is in good agreement with the values based on Sanger validation for this sample.

## Discussion

We have described a new approach to assess the accuracy of variant calls from NGS studies. The genotyping accuracy for variant calls, that is genotypes that differ from the true sample sequence, has been estimated in large-scale NGS-based projects such as the 1000 genomes project [19] or the exome sequencing project [21] and comparisons of NGS platforms [33]. In these projects, samples were sequenced to a very high mean coverage on different sequencing platforms and the reported variants represent an intersection of technical replicates and independent analysis pipelines. Even in these high quality data sets, up to about 2% of the variants cannot be validated if re-sequenced by a complementary approach such as ABI Sanger. In such a high quality exome, one detects around 15,000 single nucleotide variants per 30 Mb coding sequence and approximately 300 of them are likely false positive calls, which corresponds to an error rate of 300/30*10^6 ^= 0.00001.

Based on simulated accuracy groups for variant calls, we were able to assess the quality of an exome test sample without detailed knowledge of the applied enrichment and sequencing technology or of the bioinformatics pipeline that was used to align the reads and call the genotypes. The SDS is therefore suitable for a comprehensive quality control in all exome-based mutation screens and might turn out especially useful as a criterion for data inclusion in studies that combine exome data of different sources, due to its platform independence. The estimated genotyping error in particular might serve as quality criterion before variants detected in an exome are further analyzed: only if the estimated error is comparable to that of a high quality reference set such as the 1000 genomes project would one proceed with variant analysis. We envision that our approach to estimate the genotyping accuracy of exomes will facilitate the quality assessment of NGS data.

Software that computes the SDS, visualizes the distances to data of the 1000 genomes project, and predicts the genotyping accuracy is available for download and as a web service at GeneTalk [19].

## Web resources

The URLs for data and methods presented herein are as follows:

NHLBI Exome Sequencing Project (ESP) Exome Variant Server, http://evs.gs.washington.edu/EVS/

GeneTalk, https://gene-talk.de/qc

ftp server of the 1000 genomes project, ftp://ftp.1000genomes.ebi.ac.uk./vol1/ftp/

## Abbreviations

bp: base pair; CCDS: consensus coding sequence; CEU: central European; dbSNP: National Center for Biotechnology Information Single Nucleotide Polymorphism Database; Kb: kilobase; Mb: megabase; MDS: multidimensional scaling; NGS: next-generation sequencing; SDS: standardized dissimilarity score; ti/tv: ratio of transitions versus transversions.

## Competing interests

The authors declare that they have no competing interests.

## Authors' contributions

VH, PNR and PMK conceived and designed the study. VH developed the original code, TK and DP implemented modifications for GeneTalk. JH contributed exome data. JS and TD supervised and contributed to the statistical analysis. VH, PNR, TD and PMK wrote the paper. All authors read and approved the final manuscript.

## Supplementary Material

Additional file 1 **Supplementary material**. Figure S1: Distribution of dissimilarities of simulated error groups for different distance metrics. The distance of simulated test samples of different error groups were to the reference set was measured with an unweighted hamming metric and a genotype frequency weighted metric. The variance of the dissimilarities of the test samples is smaller in the genotype frequency weighted metric and thus allows a more precise prediction of the error group. Figure S2: Visualization of distances and estimation of error rates for different target regions. The distances of two test samples (sample 1 of high and sample 2 of medium quality) to the reference data were computed for five different target regions that differ in size. The CCDS exome comprises 29 Mb, the human phenotype ontology (HPO) [34] panel contains all exons of genes associated with phenotypic features (5.8 Mb), the Kingsmore panel comprising 548 genes of known inherited diseases (1.2 Mb) [35], all coding exons of chromosome 22 (600 kb), and the GPI panel that contains all genes involved in the GPI-anchor synthesis (45 kb). The larger the target region, the higher the number of sequence variants for comparison. This increases the precision of the estimation of the error rates. With decreasing size of the target region, the confidence intervals of the reference curve for the standardized dissimilarity score widen. While the different error rates of sample 1 and 2 can be clearly estimated and visualized for the larger target regions, gene panels below 1 Mb do not allow this assessment any more due to the larger confidence intervals. Figure S3: Data visualization techniques. Comparison of ordination methods for the visualization of the distances of exome genotypes of two test samples and high quality reference samples of a matched background population. The mean distance of test sample 2 with the low genotyping accuracy to the reference samples is larger compared to sample 1 with the high genotyping accuracy for all visualization methods. For principal component analysis and metric MDS, a substructure in the reference samples is visible that is specific to the sequencing platform. Figure S4: Exomes of different ethnicities (European (CEU), Yorubian (YRI) and Japanese (JPT)) form distinct clusters based on their similarity. For a test sample the closest cluster from the 1000 genomes project data is chosen as reference set. Figure S5: The distance of a test sample of the Yorubian reference set increases for a growing simulated error rate. Figure S6: Influence of sequencing platform on error prediction. In contrast to non-metric MDS visualization, principal component analysis of the similarities of European samples of the 1000 genomes project reveals some information about the sequencing platform that was used (A). However, the effect of the sequencing platform for predicting the genotyping accuracy is small. The predicted error rates of the test samples are comparable if the reference set is restricted to specific sequencing platforms (B, C). Table S1: Comparison of different parameters for quality assessment. Short sequence reads of test sample 1 and 2 and a sample from the 1000 genomes reference set, NA06986, were sampled to comparable mean coverage over the target region. The total number of variant calls decreases with a decreasing coverage indicating an increasing false negative error rate. Also the mean genotype quality scores decrease with a decreasing coverage indicating an increasing false positive error rate. The ti/tv ratio and the ratio of variants that are present in dbSNP vary only very little with changing coverage. Different priors in the genotyping models of Samtools and GATK result in different mean genotype quality scores for the same alignments. Quality score recalibration with GATK VariantRecalibrator performed on Samtools- and GATK-called variants ads an adjusted quality score, VQSLOD (log odds ratio of being a true variant versus being false under a trained gaussian mixture model). This score is used in GATK ApplyRecalibration to generate a tranche file of highly confidential calls. The percentage of these calls which passed as high-confidential shows an irregular behavior with respect to the mean coverage. The SDS, which is computed for the entire variant call set of a sample, correlates with its accuracy and allows a sample-to-sample comparison. Click here for file

## References

[B1] Bamshad MJ Ng SB Bigham AW Tabor HK Emond MJ Nickerson DA Shendure J Exome sequencing as a tool for Mendelian disease gene discovery. Nat Rev Genet 2011 5 11 745 755 10.1038/nrg3031 21946919

[B2] Ku CS Cooper DN Polychronakos C Naidoo N Wu M Soong R Exome sequencing: dual role as a discovery and diagnostic tool. Ann Neurol 2012 5 1 5 14 10.1002/ana.22647 22275248

[B3] Sulonen AM Ellonen P Almusa H Lepisto M Eldfors S Hannula S Miettinen T Tyynismaa H Salo P Heckman C Comparison of solution-based exome capture methods for next generation sequencing. Genome biology 2011 5 9 R94 10.1186/gb-2011-12-9-r94 21955854 PMC3308057

[B4] Clark MJ Chen R Lam HY Karczewski KJ Chen R Euskirchen G Butte AJ Snyder M Performance comparison of exome DNA sequencing technologies. Nature biotechnology 2011 5 10 908 914 10.1038/nbt.1975 21947028 PMC4127531

[B5] Holtgrewe M Emde AK Weese D Reinert K A novel and well-defined benchmarking method for second generation read mapping. BMC Bioinformatics 2011 5 210 10.1186/1471-2105-12-210 21615913 PMC3128034

[B6] Ruffalo M LaFramboise T Koyuturk M Comparative analysis of algorithms for next-generation sequencing read alignment. Bioinformatics 2011 5 20 2790 2796 10.1093/bioinformatics/btr477 21856737

[B7] Goya R Sun MG Morin RD Leung G Ha G Wiegand KC Senz J Crisan A Marra MA Hirst M SNVMix: predicting single nucleotide variants from next-generation sequencing of tumors. Bioinformatics 2010 5 6 730 736 10.1093/bioinformatics/btq040 20130035 PMC2832826

[B8] Koboldt DC Zhang Q Larson DE Shen D McLellan MD Lin L Miller CA Mardis ER Ding L Wilson RK VarScan 2: somatic mutation and copy number alteration discovery in cancer by exome sequencing. Genome Res 2012 5 3 568 576 10.1101/gr.129684.111 22300766 PMC3290792

[B9] Li H Ruan J Durbin R Mapping short DNA sequencing reads and calling variants using mapping quality scores. Genome Res 2008 5 11 1851 1858 10.1101/gr.078212.108 18714091 PMC2577856

[B10] McKenna A Hanna M Banks E Sivachenko A Cibulskis K Kernytsky A Garimella K Altshuler D Gabriel S Daly M The Genome Analysis Toolkit: a MapReduce framework for analyzing next-generation DNA sequencing data. Genome Res 2010 5 9 1297 1303 10.1101/gr.107524.110 20644199 PMC2928508

[B11] Wei Z Wang W Hu P Lyon GJ Hakonarson H SNVer: a statistical tool for variant calling in analysis of pooled or individual next-generation sequencing data. Nucleic acids research 2011 5 19 e132 10.1093/nar/gkr599 21813454 PMC3201884

[B12] Mardis ER Next-generation DNA sequencing methods. Annu Rev Genomics Hum Genet 2008 5 387 402 10.1146/annurev.genom.9.081307.164359 18576944

[B13] Pruitt KD Harrow J Harte RA Wallin C Diekhans M Maglott DR Searle S Farrell CM Loveland JE Ruef BJ The consensus coding sequence (CCDS) project: Identifying a common protein-coding gene set for the human and mouse genomes. Genome Res 2009 5 7 1316 1323 10.1101/gr.080531.108 19498102 PMC2704439

[B14] Heinrich V Stange J Dickhaus T Imkeller P Kruger U Bauer S Mundlos S Robinson PN Hecht J Krawitz PM The allele distribution in next-generation sequencing data sets is accurately described as the result of a stochastic branching process. Nucleic acids research 2012 5 6 2426 2431 10.1093/nar/gkr1073 22127862 PMC3315291

[B15] Sherry ST Ward MH Kholodov M Baker J Phan L Smigielski EM Sirotkin K dbSNP: the NCBI database of genetic variation. Nucleic acids research 2001 5 1 308 311 10.1093/nar/29.1.308 11125122 PMC29783

[B16] Bainbridge MN Wang M Wu Y Newsham I Muzny DM Jefferies JL Albert TJ Burgess DL Gibbs RA Targeted enrichment beyond the consensus coding DNA sequence exome reveals exons with higher variant densities. Genome biology 2011 5 7 R68 10.1186/gb-2011-12-7-r68 21787409 PMC3218830

[B17] Nothnagel M Herrmann A Wolf A Schreiber S Platzer M Siebert R Krawczak M Hampe J Technology-specific error signatures in the 1000 Genomes Project data. Hum Genet 2011 5 4 505 516 10.1007/s00439-011-0971-3 21344269

[B18] O'Rawe J Guangqing S Wang W Hu J Bodily P Tian L Hakonarson H Johnson E Wei Z Jiang T Low concordance of multiple variant-calling pipelines: practical implications for exome and genome sequencing. Genome medicine 2013 5 3 28 10.1186/gm432 23537139 PMC3706896

[B19] A map of human genome variation from population-scale sequencing. Nature 2010 5 7319 1061 1073 10.1038/nature09534 20981092 PMC3042601

[B20] de Ligt J Willemsen MH van Bon BW Kleefstra T Yntema HG Kroes T Vulto-van Silfhout AT Koolen DA de Vries P Gilissen C Diagnostic exome sequencing in persons with severe intellectual disability. The New England journal of medicine 2012 5 20 1921 1929 10.1056/NEJMoa1206524 23033978

[B21] Tennessen JA Bigham AW O'Connor TD Fu W Kenny EE Gravel S McGee S Do R Liu X Jun G Evolution and functional impact of rare coding variation from deep sequencing of human exomes. Science 2012 5 6090 64 69 10.1126/science.1219240 22604720 PMC3708544

[B22] Li H Durbin R Fast and accurate short read alignment with Burrows-Wheeler transform. Bioinformatics 2009 5 14 1754 1760 10.1093/bioinformatics/btp324 19451168 PMC2705234

[B23] Li H A statistical framework for SNP calling, mutation discovery, association mapping and population genetical parameter estimation from sequencing data. Bioinformatics 2011 5 21 2987 2993 10.1093/bioinformatics/btr509 21903627 PMC3198575

[B24] Danecek P Auton A Abecasis G Albers CA Banks E DePristo MA Handsaker RE Lunter G Marth GT Sherry ST The variant call format and VCFtools. Bioinformatics 2011 5 15 2156 2158 10.1093/bioinformatics/btr330 21653522 PMC3137218

[B25] Venables RBD WN Modern Applied Statistics with S 2002 Springer

[B26] Schneider TD Information content of individual genetic sequences. Journal of theoretical biology 1997 5 4 427 441 10.1006/jtbi.1997.0540 9446751

[B27] Shannon CE A Mathematical Theory of Communication. At&T Tech J 1948 5 4 623 656

[B28] Kruskal JB Nonmetric Multidimensional-Scaling - a Numerical-Method. Psychometrika 1964 5 2 115 129 10.1007/BF02289694

[B29] Jombart T Pontier D Dufour AB Genetic markers in the playground of multivariate analysis. Heredity 2009 5 4 330 341 10.1038/hdy.2008.130 19156164

[B30] Lessa EP Multidimensional-Analysis of Geographic Genetic-Structure. Syst Zool 1990 5 3 242 252 10.2307/2992184

[B31] Wang CL Szpiech ZA Degnan JH Jakobsson M Pemberton TJ Hardy JA Singleton AB Rosenberg NA Comparing Spatial Maps of Human Population-Genetic Variation Using Procrustes Analysis. Stat Appl Genet Mol 2010 5 1 10.2202/1544-6115.1493 PMC2861313 20196748

[B32] Benjamini Y Speed TP Summarizing and correcting the GC content bias in high-throughput sequencing. Nucleic acids research 2012 5 10 e72 10.1093/nar/gks001 22323520 PMC3378858

[B33] Lam HY Clark MJ Chen R Chen R Natsoulis G O'Huallachain M Dewey FE Habegger L Ashley EA Gerstein MB Performance comparison of whole-genome sequencing platforms. Nature biotechnology 2012 5 1 78 82 10.1038/nbt.2065 PMC4076012 22178993

[B34] Robinson PN Kohler S Bauer S Seelow D Horn D Mundlos S The Human Phenotype Ontology: a tool for annotating and analyzing human hereditary disease. American journal of human genetics 2008 5 5 610 615 10.1016/j.ajhg.2008.09.017 18950739 PMC2668030

[B35] Bell CJ Dinwiddie DL Miller NA Hateley SL Ganusova EE Mudge J Langley RJ Zhang L Lee CC Schilkey FD Carrier testing for severe childhood recessive diseases by next-generation sequencing. Science translational medicine 2011 5 65 65ra64 10.1126/scitranslmed.3001756 PMC3740116 21228398

