# Supplementary material for: Estimating exome genotyping accuracy by comparing to data from large scale sequencing projects
Source: Genome Med. 2013 Jul 31;5(7):69. doi: 10.1186/gm473 (PMC3978951; doi:10.1186/gm473)
Supplement: Additional file 1 — Supplementary material. Figure S1: Distribution of dissimilarities of simulated error groups for different distance metrics. The distance of simulated test samples of different error groups were to the reference set was measured with an unweighted hamming metric and a genotype frequency weighted metric. The variance of the dissimilarities of the test samples is smaller in the genotype frequency weighted metric and thus allows a more precise prediction of the error group. Figure S2: Visualization of distances and estimation of error rates for different target regions. The distances of two test samples (sample 1 of high and sample 2 of medium quality) to the reference data were computed for five different target regions that differ in size. The CCDS exome comprises 29 Mb, the human phenotype ontology (HPO) [34] panel contains all exons of genes associated with phenotypic features (5.8 Mb), the Kingsmore panel comprising 548 genes of known inherited diseases (1.2 Mb) [35], all coding exons of chromosome 22 (600 kb), and the GPI panel that contains all genes involved in the GPI-anchor synthesis (45 kb). The larger the target region, the higher the number of sequence variants for comparison. This increases the precision of the estimation of the error rates. With decreasing size of the target region, the confidence intervals of the reference curve for the standardized dissimilarity score widen. While the different error rates of sample 1 and 2 can be clearly estimated and visualized for the larger target regions, gene panels below 1 Mb do not allow this assessment any more due to the larger confidence intervals. Figure S3: Data visualization techniques. Comparison of ordination methods for the visualization of the distances of exome genotypes of two test samples and high quality reference samples of a matched background population. The mean distance of test sample 2 with the low genotyping accuracy to the reference samples is larger compared to sample 1 with the high geno [file gm473-S1.DOCX]

Supplemental Data

Estimating Exome Genotyping Accuracy by Comparing to Data from Large Scale Sequencing Projects

Verena Heinrich, Tom Kamphans, Jens Stange, Dmitri Parkhomchuk, Thorsten Dickhaus, Peter N. Robinson, Peter M. Krawitz

Figure S1: Comparison of the variance for the mean distances of simulated accuracy groups for hamming distance and a genotype frequency weighted metric. The smaller variance for the genotype frequency weighted similarity metric improves the prediction of the genotyping accuracy.

|  | Figure S2: visualization of distances and estimation of error rates for different target regions. The distances of two test samples (sample 1 of high and sample 2 of medium quality) to the reference data were computed for five different target regions that differ in size. The CCDS exome comprises 29Mb, human phenotype ontology (HPO) [34] panel contains all exons of genes associated with phenotypic features (5.8Mb), the Kingsmore panel comprising 548 genes of known inherited diseases (1.2Mb) [35], all coding exons of chromosome 22 (600 kb), and the GPI panel that contains all genes involved in the GPI-anchor synthesis (45kb). The larger the target region, the higher is the number of sequence variants for comparison. This increases the precision of the estimation of the error rates. With decreasing size of the target region the confidence intervals of the reference curve for the standardized dissimilarity score widen. While the different error rates of sample 1 and 2 can be clearly estimated and visualized for the larger target regions, gene panels below 1MB do not allow this assessment any more due to the larger confidence intervals. |
| --- | --- |

Figure S3: Data visualization techniques. Comparison of ordination methods for the visualization of the distances of exome genotypes of two test samples and high quality reference samples of a matched background population. The mean distance of test sample 2 with the low genotyping accuracy to the reference samples is larger compared to sample 1 with the high genotyping accuracy for all visualization methods. For PCA and metric MDS a substructure in the reference samples is visible that is specific to the sequencing platform.

Figure S4: Exomes of different ethnicities (European CEU, Yorubian YRI, Japanese JPT) form distinct clusters based on their similarity. For a test sample the closest cluster from the 1000 genomes project data is chosen as reference set.

Figure S5: The distance of a test sample of the Yorubian reference set increases for a growing simulated error rate.


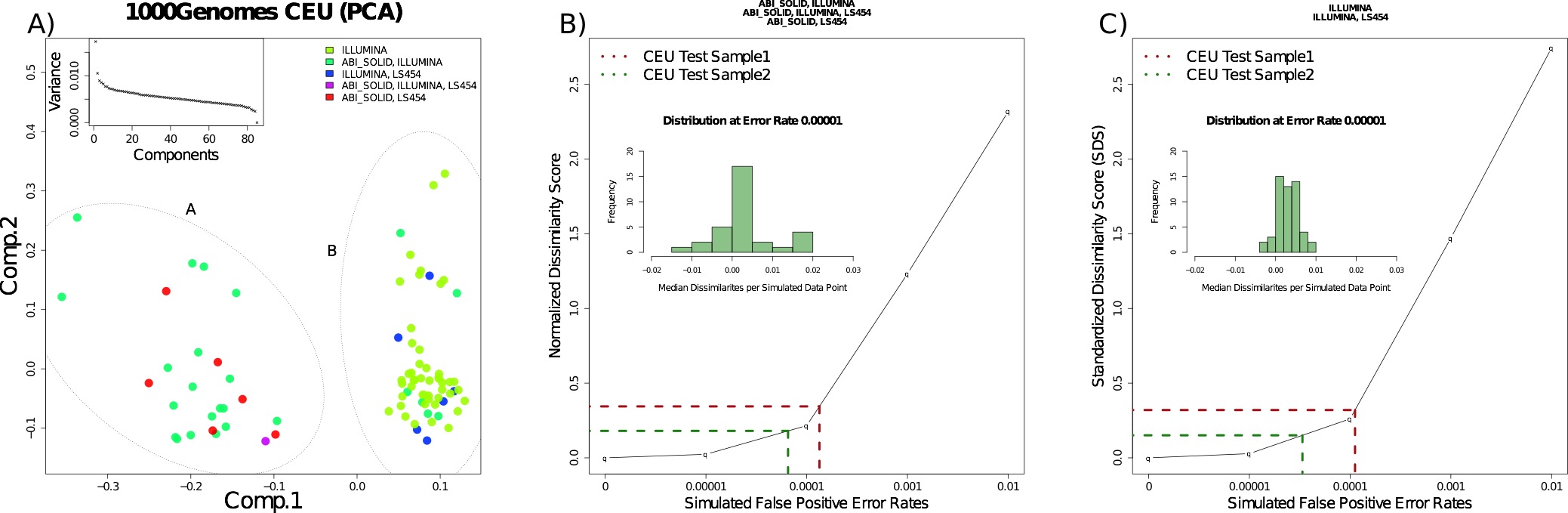


Figure S6: Influence of sequencing platform on error prediction. In contrast to non-metric MDS visualization PCA of the similarities of European samples of the 1000 genomes project reveals some information about the sequencing platform that was used. However, the effect of the sequencing platform for predicting the genotyping accuracy is small. The predicted error rates of the test samples are comparable if the reference set is restricted to specific sequencing platforms.
